# Learning to hunt Crocodiles: social organization in the process of knowledge generation and the emergence of management practices among Mayan of Mexico

**DOI:** 10.1186/1746-4269-9-35

**Published:** 2013-05-24

**Authors:** Fernando Zamudio, Eduardo Bello-Baltazar, Erin IJ Estrada-Lugo

**Affiliations:** 1 Grupo de Etnobiología, Instituto de Biología Subtropical – sede Iguazú, Facultad de Ciencias Forestales, Univ. Nac. de Misiones and Asociación Civil Centro de Investigaciones del Bosque Atlántico (CeIBA), Bertoni 85, 3370, Puerto Iguazú, Misiones, Argentina; 2 Departamento de Gestión Comunitaria de los Recursos Naturales, El Colegio de la Frontera Sur-Unidad San Cristóbal de las Casas Chiapas- Ap. 63, San Cristóbal de Las Casas, Chiapas 29290, Mexico

**Keywords:** Social organization, Learning, Local ecological knowledge, Mayan, Commercial hunting, Common resources, *Crocodylus moreletii*

## Abstract

**Background:**

New kinds of knowledge, usage patterns and management strategies of natural resources emerge in local communities as a way of coping with uncertainty in a changing world. Studying how human groups adapt and create new livelihoods strategies are important research topics for creating policies in natural resources management. Here, we study the adoption and development of *lagartos* (*Crocodylus moreletii*) commercial hunting by Mayan people from a communal land in Quintana Roo state. Two questions guided our work: how did the Mayan learn to hunt *lagartos*? And how, and in what context, did knowledge and management practices emerge? We believe that social structures, knowledge and preexisting skills facilitate the hunting learning process, but *lagarto* ecological knowledge and organizational practice were developed in a “learning by doing” process.

**Methods:**

We conducted free, semi-structured and in-depth interviews over 17 prestigious *lagartos* hunters who reconstructed the activity through oral history. Then, we analyzed the sources of information and routes of learning and investigated the role of previous knowledge and social organization in the development of this novel activity. Finally, we discussed the emergence of hunting in relation to the characteristic of natural resource and the tenure system.

**Results:**

*Lagarto* hunting for skin selling was a short-term activity, which represented an alternative source of money for some Mayans known as *lagarteros*. They acquired different types of knowledge and skills through various sources of experience (individual practice, or from foreign hunters and other Mayan hunters). The developed management system involved a set of local knowledge about *lagartos* ecology and a social organization structure that was then articulated in the formation of “working groups” with particular hunting locations (*rumbos* and *trabajaderos*), rotation strategies and collaboration among them. Access rules and regulations identified were in an incipient state of development and were little documented.

**Conclusions:**

In agreement to the hypothesis proposed, the Mayan used multiple learning paths to develop a new activity: the *lagarto* hunting. On the one hand, they used their traditional social organization structure as well as their culturally inherited knowledge. On the other hand, they acquired new ecological knowledge of the species in a learning-by-doing process, together with the use of other sources of external information.

The formation of working groups, the exchange of information and the administration of hunting locations are similar to other productive activities and livelihood practiced by these Mayan. Skills such as preparing skins and *lagartos* ecological knowledge were acquired by foreign hunters and during hunting practice, respectively. We detected a feedback between local ecological knowledge and social organization, which in turn promoted the emergence of Mayan hunting management practices.

## Background

In the context of contemporary rural realities, characterized by economic and environmental changes, a new kind of knowledge, use patterns and management strategies of natural resources have emerged as a way of coping with change and uncertainty [1-3]. Studying how conservation and management practices have evolved, and how knowledge is created, changed and used, are important research topics for management and natural resources policies [4]. Likewise, through this approach the mechanisms of learning involved in the development of economic activities, which ultimately refer to the adaptation of groups to new scenarios, can be studied.

The construction of ecological knowledge in non-Western societies with oral tradition, involves a lengthy process of observation and feedback with the environment [5]. Learning about ecological dynamics and skills for survival, as in other domains has been in large part incremental and cumulative [6]. Learning is shaped by two processes, cultural transmission on the one hand and acquisition of knowledge in practice or “learning by doing” on the other [7,8]. Although cultural transmission, especially among family members is considered one of the most conservative mechanisms of knowledge [8], different cultures have developed their own interpretations of the learning process. In turn, these have been useful to reinterpret the results of other related processes such as the emergence of knowledge and management practices. For example, for the Anishinaabe of Canada learning involves journeying along the land where the places have memories that are constantly transmitted and where new ones are created [9].

Traditional or local ecological knowledge is one mayor force involved in natural resource management in consumptive activities like hunting, fishing or gathering. Knowledge about distribution, abundance and behavior concerning resources and characteristics of landscape are the principal source of information for decision-making about where, when and how to harvest animal or plants [5,10-12]. The extent of knowledge enables individuals to maximize harvest success, for example, through spatial and temporal segregation of the exploitation spot (“rest” concept), communication (exchange of information), competition (secrecy and deceptions) and development of social norms [5,10,13].

Communication and collaboration among users is a valuable mechanism to interchange relevant information and knowledge regarding resources, both in traditional groups of hunter-gatherer [11,12] and in high-technology fisheries [13,14]. Exchange of information is the common way of learning from others in most of these cases. Also, it has been observed that the interconnection between rules and decision-making process promotes knowledge generation [11].

In the development of new productive activities knowledge and practices may take time to develop. However, some study cases suggest that preexisting social structures or social networks may accelerate the learning process see [2]. Knowledge developed in this process can be based on knowledge and skills acquired *a priori* by enculturation models [8] but local ecological knowledge is often gained more recently over the lifetime of individuals [15].

This paper addresses the question of how new knowledge and practices have emerged from *lagarto*s (*Crocodylus moreletii*) commercial hunting practiced in the past (between 1960–1980) by Mayan peoples from a communal land (*ejido*) in Quintana Roo state.

International and national demand of crocodile skin enhanced hunting of these reptiles in all the Mexican territory, and in large part of the crocodilians distribution around the world [16]. Reptiles are food and medicinal resources widely used among local people in both commercial and subsistence activities, while indiscriminate use endangers species conservation [17-20]. Given the economic and cultural importance of reptiles for various human groups is necessary to pay more attention to the development of sustainable management plans for species use [21]. An important step in this direction is to understand the cultural, social and traditional roles of the fauna in each local context [22].

The case study analyzed meets a set of characteristics which are different from other Maya’s traditional activities. Mayan *lagarto* hunting was; a) a purely economic activity, as its flesh is considered unfit for consumption; b), traditionally *lagartos* were not subject to hunting because of the latter; c) the activity was performed by the Mayan for a period of less than 10 years (boom-bust activity) as a result of the influence of markets and then prohibited after the total ban on hunting proclaimed by the Mexican state; d) it was developed over a common resource and under open access regime (State lands) [23,24]. Ecological knowledge generated by hunters during the activity is considered complementary to a *lagartos* population sampling conducted in communal lands. It provides information on the habitat and behavior of *lagartos* little explored by scientists [24].

Two questions guided our work: how did the Mayan learn to hunt *lagartos*? And how did, and in what context, knowledge and management practices emerge? To answer these questions we analyzed the sources of information and routes of learning as well as mechanisms involved in the acquisition of knowledge. We hypothesize that preexisting social structures, knowledge and skills facilitate the hunting learning process but *lagarto* ecological knowledge and organizational practice were developed in a “learning by doing” process.

Also, we investigated the role of previous knowledge and forms of social organization used by the Mayan in the development of this new activity. Finally, to analyze the context in which a management system has appeared, we discussed the emergence of hunting in relation to the characteristic of natural resource and the tenure system in the framework of the literature referred to common resources.

## Methods

### Study site

**Figure 1:**
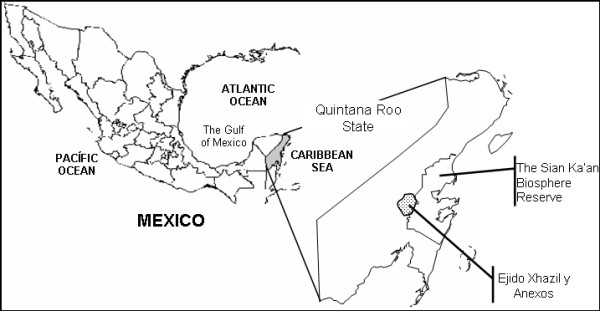
**Map of the *ejido *of *Xhazil y Anexos *and of the Reserve of the Biosphere of Sian Ka` an, the main Maya hunting area.**

This study was performed in the Mayan *ejido* of *Xhazil y Anexos* in Quintana Roo, Mexico (Figure 1). The 54,000 Ha *ejido* consists of three communities, *Chancah Veracruz*, *Xhazil Sur* and *Uh May* which are located 3–6 km one from the other (henceforth called *Xhazil*). They are Mayan-Yucatec people with historical presence in the region and today speaking both Spanish and Mayan. These Mayan are descendants of rebels who fought in the so-called *guerra de castas* (caste war) in the 19th century [25].

These communities practice *milpa* (polyculture of maize or shifting cultivation), garden cultivations, wildlife hunting, fishing and use a wide variety of resources for subsistence (plants, honey among others) [26]. The extraction of *Manikara zapota* gum was a relevant activity in the past that still stands at a low level in some families. At the present time the most important economic activity is logging of valuable tropical woods [27].

The *ejido* covers areas of semi-deciduous and semi-evergreen forest, sawgrass marshes or *savannas* dominated by *Cladium jamaicensis* and water bodies as lagoons and sinkholes [27]. The region has a warm subhumid climate with an annual rainfall of 1,100 to 1,200 mm and an annual average temperature of 26°C. This allows a marked rainfall pattern of drought from December to May.

*Lagartos* hunting took place mainly outside the *ejido* of *Xhazil* in a vast wetland in the surroundings. Years after the hunting period, in 1986, the majority of the old hunting locations were included in the Sian Ka’an Biosphere Reserve [28]. This is the second more extensive wetland in Mexico with 528,000 ha (Figure 1). Hunting was practiced in a flood plain made up of sawgrass marshes and dwarf mangrove (*Rhizophora mangle*, *Laguncularia racemosa*, among other species) locally called *savanna*. In this landscape, *petenes* or tree islands that elevate on the flood plain are common [29]. *Petenes* can be either monospecific (e.g. *R. mangle*) or have a semi-evergreen forest composition; those of a larger size can even contain fresh water or a sinkhole inside [29,30].

### Data collection and analyses

Fieldwork included preliminary visits and stays at communities where the research team had worked since 2000. Stays at the *ejido* lasted 20 days a month for 5 months (from January to May 2004). Through informants identified in previous works and with the snowball technique [25], 17 key informants were selected among the three communities (only males); most of these informants are recognized as prestigious *lagarto* hunters. They ranged from 54 to 83 years old, having practiced this activity for 5 to 10 years, and represented more than 80 percent of the total number of hunters alive. The distinction made in the body text between types of hunters (*lagarteros* versus other Mayans hunters) emerged from the investigation, regardless of the consideration of all respondents as key informants.

Because the activity was carried out in the past, the hunters’ oral history was taken into account during the visits. Free, semi-structured, and in-depth interviews were conducted (a total of 50) following Bernard protocols [25]. The topics discussed in the interviews were behavior and ecology of *lagartos*, local practices and hunting strategies used and local organization. As new information emerged from the interviews or from field observations, it was subject to the consideration of hunters in new visits, giving rise to continuous feedback. This allowed us to assess the individuality or generality of statements or facts. In this respect we visited several wetlands within the *ejido* and in the limit of the ancient hunting places. These journeys allowed us to bring about relevant topics of conversation which otherwise would not have arisen. Queries to the hunters about characteristics of a specific wetland (e.g. sinkhole called “Buluha”) or observations made in wetlands, fostered vivid memories among those interviewed [24]. We also participated in other currently performed activities as fishing, hunting of other animals and agricultural work.

For the interviews, we used a notebook and a tape recorder, as well as maps and aerial photographs of the old hunting area. The information obtained is qualitative and follows the methodological protocols proposed by Johannes *et al*. [31] and Davis and Wagner [32]. These authors consider the selection of “expert” informants adequate, in contrast to a random selection, and the usage of less formal interviews that allow guiding the interviewer to more relevant topics in the context of the activity under study. A composed tabs database was elaborated using Microsoft Access (900 tabs); this was ordered according to general topics (for example; hunting practices) and specific topics (for example; sawgrass burning) which permitted cross-checking information according to informants, community and specific topics. In this way, it was possible to grasp a collective view of the activity as a result of the combined answers of the group of informants and complementary activities developed during the investigation.

In order to calculate the number of hunted crocodiles we averaged the number of animals killed in a “bad” and “good” hunting day (minimum and maximum) from respondents who provided data about both of them. In the same way we calculated the frequency of hunting trips and how long they lasted.

## Results

### Emergence of *lagarto* hunting

*Lagarto* hunting became a new activity for the Mayan at the *ejido* of *Xhazil* as a way of obtaining money through its skin commercialization, it was stimulated by traders and foreign hunters who arrived in the region attracted by the presence of large wetlands. Hunting was performed freely in a vast hardly accessible public wetland (fiscal lands) located in the *ejido* east border, where people of diverse geographical and cultural origin merged in the same hunting place. Encounters between groups of hunters in the *savanna* or traces of the hunting activity as human footprints or vultures flying around skinned animals, were commonly referred to by interviewed hunters, denoting the intensity of the activity.

According to people interviewed, they hunted on foot during the drought season highest peak (February-May), which allowed them to explore the *savanna* exhaustively. The burning of sawgrass vegetation was a common practice that favored walking in search of *lagartos* footprints. In contrast, foreign hunters hunted in any season and generally used boats that enabled them to enter flooded areas.

For the Mayan, hunting *lagartos* was considered an “annoying”, “dirty” activity and as a result a job “only for some people” due to the drudgery of the activity (long distances, swampy soil and hazard). While respondents indicated that many Mayan ventured for some time in a hunting journey, only a few were “devoted” to it or “true *lagarteros*”. This internal distinction made by respondents reflects two different production strategies based on the frequency with which the hunting took place and on an efficiency factor that distinguished *lagarteros* from the rest of Mayan hunters (Table 1). The strategy of the *lagarteros* was to maximize the catches along the period of *lagartos* hunting in the dry season. After a hunting trip, hunters returned to sell their skins to intermediaries and immediately afterwards got provisions to return to the *savanna* to search for more *lagartos*. Some of these Mayan even hired other people to work in their agricultural plots during this time delegating one of the most important productive activities for the four months the hunting activity lasted. Instead, occasional hunters performed from 2 to 6 hunting trips a year for occasional cash needs, “when there was no money or work, we would get to hunt *lagartos* to make a few bucks” (Table 1).

**Table 1:** Typology of Mayan hunters according to the workflow or time dedicated to the activity

	***Lagarteros *(N = 5)**	**Occasional hunters (N = 9)**
# years made activity	From 5 to 10	From 2 to 4
Frequency (hunting trips/year)	From 8 to 12	From 2 to 6
# people per group	From 3 to 4	From 3 to 4
# days of hunting	From 3 to 5	From 7 to 15
Average hunted *lagartos*/day	5.3 (min)	2.56 (min)
	12.6 (max)	4.89 (max)
Average hunted *lagartos*/year/grup	127.2 (min)	35.8 (min)
	756 (max)	440 (max)
**Overall average hunted *lagartos*/year/grup**	**441.6**	**237.9**

Shows maximum (max) and minimum (min) number of *lagartos* hunted according to the Mayan in the period between February and May.

In the accounts of both types of hunters, however, there is a common concept of efficiency that was related to four variables: 1) hunters knowledge and skills to walk to hunting places in a wetland of difficult access and scarce visibility (highly vegetated sawgrass vegetation in a monotonous and flat landscape), 2) knowledge of *lagartos* behavior and distribution 3) the skills to hunt *lagartos* and skinning them and 4) an efficient organization among small groups of hunters (see below).

Hunters remarked that by the time the activity was close to its end the abundance and, especially the size of the hunted *lagartos,* decreased. However, for most hunters, *lagartos* were an unlimited resource due to their high abundance, the size of the wetland where they hunted, and the fact that they did not have access to the muddiest or the most hazardous sites. Moreover, according the Mayan big *lagartos* were more cantankerous and avoided hunters.

### Sources of knowledge and learning

Professional foreign hunters from different Mexican states and even from Belize (a bordering country) were pioneers and promoters of the activity in the area. These hunters hired the Mayan from *Xhazil* as helpers and guides for hunting trips in the *savanna* before they started the activity formally. The Mayan learned some hunting techniques from these foreign hunters, such as the way of using harpoons or skinning and drying skin. However, there is evidence of a learning process during the practice itself in the speech of the interviewees. Hunters reported that “walking and working are all learned … at the beginning we saw it difficult and did not hunt a lot, but after five trips we already knew how to do it” (F.C.) or comments such as “[after guiding foreign hunters] we saw how and where to do it and we started practicing it…” (L.Y.).

The hunting of *lagartos* among the Mayan emerged as a group activity that was changing as the hunting trips extended, accounting for the above mentioned learning process. Groups of between 6 to 8 people that explored the *savanna* and even went to the sea (more than 40 km from the communities) in search of *lagartos* gathered for the early hunting trips. Later the group number decreased to 3 or 4 people as the hunting effort in big groups was unproductive in terms of cost-benefit. Both coastal environments and the *savanna* were places little explored by the Mayan until this time.

**Figure 2:**
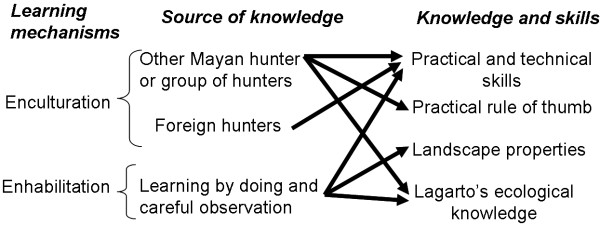
Sources of knowledge and skills acquired throughout different learning mechanisms.

On the other hand exchange of information and knowledge among groups of hunters was a common practice of cooperation between the Mayan (see below) which influenced the transmission of practical and technical skills and practical rules, as well as *lagartos* ecological knowledge. In this learning context, the Mayan gained different types of knowledge and skills through various sources (Figure 2). Among them, we identified the knowledge gained from individual practice (acquired through learning by doing and careful observation), from foreign hunters and from other Mayan hunter or group of hunters.

### Components and management principles

The management system developed by the Mayan is composed by a set of local ecological knowledge about the *lagartos* ecology as well as landscape properties and dynamics, a social organization structure, and although just outlined, a set of rules on the activity access and regulation (Table 2).

**Table 2:** **Components and management principles of Mayan *lagarto *hunting**

**Management components**	**Local expressions**	**Purpose and comments**
*Lagartos* distribution (LD)	“In the *savanna* there are dens, there are many […] near between 2 to 5 *mecates* [local measurement, *1 mecate* ~ 20 m2], […] it looks like a town where *lagartos* live” (A.C.)	Allows hunters to identify areas where hunting is safe and effective.
Key-hunting habitat (K-hH)	“The *lagartos* are in small pools or *pozas* in the *savanna* […] they are also in lagoons but the animals dens are is in the *pozas* and there it [the hunting] does not fail” (A.P.).	Allows hunters to minimize search time.
*Lagartos* movement dynamics (LMD)	“The *lagartos* stay in a *poza* for one or two weeks and when they get upset (*se fastidia*) they go to another one looking for food” (J.B.S.).	Allows hunters to predict the delay in occupation of this key hunting habitat dropped off by the *lagartos*.
	“Sometimes we entered to work in one place and we killed 2 or 3 *lagartos* and when we were leaving, other *lagartos* came because the houses [dens] were empty, and at night as *lagartos* were walking, looking, they arrived” (R.Y.).	
Spatial orientation skills and management practices (SkMp)	“To be able to hunt *lagartos* it is necessary to know the places they [the *lagartos*] live in, the footprints and the paths to know how to follow them […] the who does not know loses […] all work has to be worked out, may be farther away, but if the soil is firmer [for walking], is faster” (J.T.).	Allows hunters to recognize the places (surfaces) where they can walk. It promotes the creation of “mental maps” (group or individual) of key-hunting habitat.
Social organization	“If you know other hunters, they tell you where they went and you go farther away, look for another *rumbo* […] we worked in stages, it’s like a rotation, where we started we finished […] we waited until others *lagartos* arrived” (L.Y.).	Allows hunters to divide profits from huntings through cooperation among groups. The exchange of information and knowledge promotes social learning.
	“you asked where other hunter had gone and they told you; where left the Salt or in Birds [*trabajaderos* names] and according to what they told you, you went there or not” (A.Q.)	
Acces rules	“When it was burning in some place it was a sign that they were working [hunting] there and we had to find another place to go. […]” (A.Q.)	Encounters with other hunters promote flexibility in the decision-making process. Competition promotes secrecy but only in specific key hunting habitat.
	“There are some who are jealous of their hunting grounds [key hunting habitat] and did not burn so others do not know where it is” (N.C.).	
Regulation rules	“Many get upset when they see a destroyed den because [the *lagartos*] live there, it's like the *tepezcuintle* [*Aguti paca*] if you destroy the den they do not come back” (N.C.).	Underrepresented and lax rules of use. Defined by hunters and by markets.
	“We hunted animals of 7 or 8 feet, large animals, 5 feet up we hunted, not the little ones because they [the traders] did not buy” (A.P.)	

Some hunter quotations considered representative of the management system developed are cited in quotation marks. Percentages of answer frequency of hunters about management components are given. Social organization, access and regulation rules were considered qualitative variables.

*LD* – According to the hunters, *lagartos* live “in clusters” during drought time (29% of interviewed). Small islands of mangrove “*verdecitos*” (light green) and *pozas* (59% of interviewed) were mentioned as a two main habitats where they could find dens of *lagartos* in the savanna, *K-hH* – Successive hunting of the animal in the same den or place (59%), *MD* four kinds of movements made by the *lagarto* were identified, I) movements around the place occupied, such as dens, *pozas,* and mangrove islands (25%), II) movements among habitats (37.5%), III) long distance “trips” (43.75%), IV) during mating time (May), the males move from one *poza* to another until they find a female (31.25%), *SkMp* – *Tool used*: harpoon (94%) and firearms like shotguns (16 gauge or 20) or rifles (22 gauge) to a lesser extent, *Find Preys*: burning of sawgrass (65%), following trails (65%), appearance of muddy water in *pozas* (29%) and the sound of response after the imitation of *lagarto*’s vocalizations (18%), *Hunting Technique*: in dens and *pozas* consisted in sticking a long pole into the den “roof” until the animal was reached (94%), capturing the *lagarto* with a hook-bait (29%), using rafts to hunt in lagoons or sinkholes (29%).

Local ecological knowledge is focused on the distribution, habitat and behavior of *lagartos* and on the characteristics of the landscape. In the *savanna* they identified areas and habitat where *lagartos* are aggregates like “*lagartos* villages”. There, formations known as *pozas* (pools) and caves refer to places indicated as a key habitat where “there are always *lagartos*” indicating their continued presence in such formations. The logic of the practice indicates that the hunting of one *lagarto* promotes the availability of a shelter that will in turn be occupied by another *lagarto* (Table 2). This was explained by the hunters because *lagartos* “walk a lot” looking for various resources; a “house” or shelter with suitable characteristics like sufficient water and food, or a couple during the mating season. The later coincides with the drought period when there is a shortage of these resources and *lagartos* move a lot. In turn, at that time hunters could enter on foot to the *savanna* to search *lagartos*.

The dynamic of *lagartos* movement was learned by observation and inferences from footprints -the main strategy used to search *lagartos*- and as a result of the effect of observation of their own hunting in key habitat. They hunted *lagartos* repeatedly in these sites in different hunting trips both within the same season or in different ones. Thus, hunters corroborated that *lagartos* returned to empty caves (Table 2). Continuous passage by the same route, exploration ability and a notable orientation across space allowed hunters to develop “mental maps” of the places where there were dens or *pozas* in areas known by them (see below local concept called *trabajadero* and *rumbos de caza*). Precise references to specific hunting places in the territory (e.g. *pozas* or dens) were commonly mentioned by the hunters sometimes accompanied by references about the size of the hunted animal or anecdotes about the place. The location of those specific key hunting places where *lagartos* were killed “every week” was a piece of information that some hunters did not always share (concealment), retaining their exclusivity of use (See regulations rules in Table 2).

Around this knowledge the Maya developed a social organization that was expressed in the formation of work groups with hunting courses and hunting places where they “work” or hunt *lagartos*. These were locally called *rumbos de caza* and *trabajaderos* respectively. The *rumbos de caza* consisted of tracks and paths through the *savanna* leading to different *trabajaderos*. These are areas where *lagartos* were abundant and constantly present (Table 2). The *rumbos* were not used by one group of hunters exclusively but some of them were associated to family groups or groups coming from different communities (e.g. “the Cruz”- name- or “those from *Xhazil*”). On the other hand *trabajaderos* were generally marshes associated to islands of trees (*petenes*) locally called *mogotes*. These islands were appropriate places for hunters to camp and provided resources that were scarce in the *savanna,* as water to drink (petenes’ interior sinkhole), trees for shelter and firewood to cook.

The Mayan interviewed reported at least 16 *trabajaderos* which were called by names that made reference to the place characteristics or to stories related to them. For example the so called *Pucte* refers to one that had a large pucte-tree (*Bucida buceras*). The Mayan hunter used these toponyms as a geographical reference to exchange information with related or “associated” working groups, about the *rumbos* location, camps, hunting achievements and about the *trabajaderos* recently used by them or by other hunters (Table 2).

Access rules and regulations identified were in a pristine state of its development and were little documented (Table 2). The regulatory rules instead represented in one case a social punishment for those who do not take care of *lagartos* caves and in other case a rule imposed by the market over skin minimum size for sale which restricted hunting on lower age groups.

## Discussion

### Learning to hunt *lagartos*

Some Mayan of *ejido* the *Xhazil* practiced the hunting of *lagartos* in response to the foreign demand of crocodile skins, finding in this activity an opportunity to generate income, thus introducing a new activity to their production system. In this context hunting of *lagartos* can be interpreted as an adaptive change to their social-ecological system that led to a new relationship with the environment, based on learning in practice. The Mayan had made incursions in previously unexplored and inhospitable environments and learned about the dynamics of the wetland as well as the ecology of *lagartos* over a period of about 10 years or less see [24]. As a result the above mentioned new market had triggered an intense period of experimenting and rapid learning on a previously unused resource.

Similar changing situations and responses to crisis have been documented around the world showing in some cases rapid community adaptation to new circumstances [1,33]. One example is the Inuit use of bird skin in parkas manufacture after the caribou crisis, from which skins for traditional parkas were obtained [6]. Other remarkable cases are constituted by immigrants or groups of people that generated knowledge and management practices on environments which were different from their original residences in a relatively short time see [2,34]. In this respect wage labor is recognized as an important source of new knowledge which exposes people to new places, new social settings, and new productive systems which ultimately may stimulate innovation [1].

The Mayan obtained knowledge and skills from hunting *lagartos* through different sources, although the evidence we gathered indicates that *lagartos* hunting was mainly learned during hunting journeys. The frequency and intensity of hunting (frequency of hunting trips) are factors that conditioned the acquisition of ecological knowledge and practical skills. These factors determine the extent of interaction with the environment and therefore the learning opportunities. True *lagarteros* were more efficient in their hunting returns compared to those who made a few hunting trips per year. Some authors agree with this statement and remark the importance of learning opportunities in the development of knowledge on various natural domains [7,35-37]. According to Boster [38] direct experience with elements of nature is probably more important than learning by social contact. Thus, kinship networks constitute only partial channels of the flow of goods and information between people.

Nevertheless, we do not imply that learning to hunt *lagartos* is entirely an enskilling (acquisition of knowledge in practice) or an enculturation (cultural transmission) process. Instead, we would like to stress that different skills and knowledge are acquired through different learning paths simultaneously, as we originally hypothesized. For example, how quickly Mayan developed the activity may be related to the fact that hunters were at the peak of development of their hunting skills, which according to some authors is reached between 30 and 40 years See review [39]. We suggest that track detection and interpretation, the reading of environmental signals (e.g. characteristics of the soils according to vegetation) or practical rules used both in subsistence hunting or fishing were learned from other people, especially relatives, through daily traditional life .

Moreover, while some practical rules such as “checking the dens periodically” or tracking traces may have derived from the logic and skills used by the Mayan in wildlife hunting [40], following Ingold words [7] we consider that “the accomplished hunter consults the world [the nature], not representations inside his head”. Even if the rule can be transferred by other hunter, the trainee needs to “read” and interpret signs such as footprints and other traces at the cave entrance, among others, to discern if the animal is present in the cave. This suggests both individual practice and teaching of practical rules but also an “education of care” on what and how to look and interpret those signs [7,41].

On the contrary knowledge on the behavior of *lagartos* and on the *savanna* basic ecological principles was acquired firstly through personal and group experience in learning by doing, as *lagartos* were not hunted in the past. According to some authors animal behavior can be partially taught (through conversation, proverbs or histories) or explained but to be interpreted it necessarily needs to be observed and experienced in practice [39,42].

However, individual or group knowledge acquisition and the time it takes to develop must be distinguished from the ability of social-ecological systems to respond to changes. This capability is based on the presence of pre-established social structures (e.g. social networks), institutions involved in regulating rules and communication factors [2,5,9,13].

### Feedback between social organization and local ecological knowledge

**Figure 3:**
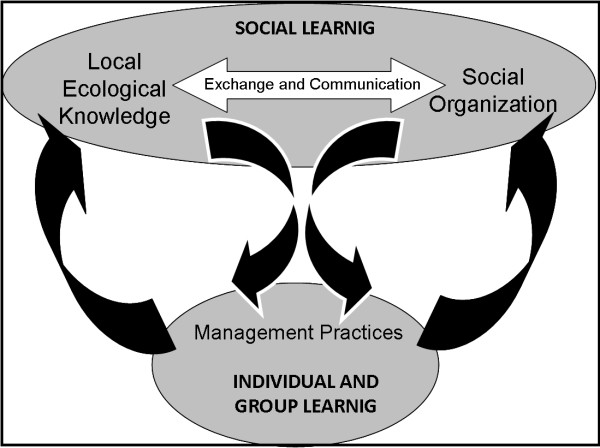
**Emergence of management practices as a product of feedback between local ecological knowledge and forms of organization.** This process is mediated by social learning in the frameworks of exchange of information and linked communication factors.

The emergence of hunting management practices among the Mayan, in our opinion, is the result of feedback between local ecological knowledge and social organization, as illustrated in Figure 3. While, the carrying out and development of management practices in the field have promoted different learning paths, these in turn, have fostered changes and additions to the *corpus* of local ecological knowledge and even in the social organization. For example, recurrent hunting of *lagartos* in the same cave promoted new insights into the knowledge about the dynamics of their movements and this experience led to changes in the conformation of hunters working groups, which were reduced in number according to the balance between costs and benefits. A similar mechanism was documented by Parlee and Berkes [43] in berry harvesting by Tetlit Gwich’in in Northern Canada. They observed a dynamic interaction between knowledge generation and decision-making. So changes in abundance and distribution of berries promoted modifications on rules of use, access to berry patches and sharing of information about the harvest among other ecological clues. As in this case, Mayan daily observations and experience gained during journeys through the *savanna* are used as sources of knowledge to restructure and change management practices (Figure 3).

As in other consumptive activities the above-mentioned mechanism, the interchange of experiences and (individual or group) knowledge, has a relevant influence in the acquisition of expertise and efficiency in *lagarto* hunting [11-13]. In North Atlantic fisheries for example receiving reliable information is the most common way of teaming and a major factor in terms of fishing capacity [13]. Besides, for the Mayan communication between groups and/or between hunters has in turn functioned as a mechanism of collaboration to share profits while allowing to avoid failure in hunting by going to places recently hunted. Simillary the practice of observation or “checking the berries” provided Teetl’it Gwich’in women insight about where and when they can find the best berries. The sharing of these observations among harvesters is also fundamental to the success of the harvest in any given year [43].

### Management system roots

Results show that *lagarto* hunting was based on existing organizational forms related to “ways of doing” and to understanding the dynamics of natural systems traditionally developed by these Mayan. The formation of working groups and the division of territory in *trabajaderos*, under the notion of rest, are represented in other productive activities developed by the Mayan as slash-burn agriculture [44].

Previous works in *Xhazil* have shown that the formation of small working groups to perform activities are a usual form of social organization to reach common goals while the definition of areas for family use, like *rumbos agricolas familiares* (family farm courses), have determined the way of space appropriation [25,45,46]. Those rituals of the agricultural and ceremonial calendar give meaning and coherence to collective activities [45]. According to Ostrom [47] previous experience with forms of local organization has greatly enhanced the repertoire of rules and strategies known by local participants whereas it is more likely that users agree upon rules the operation of which they understand from previous experience. Thus, previous social arrangements provides a shortcut to problems raised by new activities.

Moreover, behind the practice of rotation of hunting places (e.g. *trabajaderos*) there is an implied understanding on renewal cycles and the length of time that *lagartos* population or other resources would need to replenish themselves [4]. In farming this understanding reaches high levels of refinement and is related to knowledge about the characteristics of the soils and the ecological succession process of vegetation in transformed plots [44,48].

Practices related to the spatial division and rotation of areas of hunting or fishing have not been identified in previous studies in *Xhazil*[40,49-51]. But subsistence hunting of wildlife widely practiced in these communities, as noted in the previous section, was the basis for the development of *lagartos* hunting. Their daily implementation practices promoted learning about ecology and hunting techniques as well as the acquisition of physical and perceptual skills (Figure 3).

### Mayan hunting lagartos: contributions over the commons

The study case presented suggests that resource management systems can arise even in open land tenure regimes and common property resources like *lagartos*. Combination of open regimes use and market demands like in our study, often lead to resource depletion see examples in [5]. Moreover, evidence suggests that the degree of success in resource management is defined by complex interactions among the characteristics of resources, property rights and other institutional arrangements, as well as by the socio-economic context [52,53].

From an ecological point of view it has been argued that when resources are important, limited, predictable, and depletable, and they are under the control of resource harvesters, local communities more often develop ways of managing them [54]. L*agartos* were a relatively important resource only for those most dedicated hunters and an unlimited resource, while it was a complementary activity, and in some cases occasional, within the Mayan production system. Moreover, their hunting was carried out under open tenure systems, without defined norms and access rules, at least for all hunters using the *savanna* (foreign and Mayan).

As wildlife *lagartos* are a common property resource for which exclusion is difficult and joint use involves subtractability [53,55]. In this case the defense of the resource was not possible as the activity was done on large extensions of state land, but also not necessary because it was an unlimited resource according to local perception (non-depletable resource). According to Berkes [54] territoriality or resource defense occurs when the benefits of use outweigh the costs of defense and this was not the Mayan case.

On the other hand *lagartos* were a predictable resource, as they were in the same places each year. According to Ostrom [47] a highly predictable resource is much easier to understand and manage than one that is erratic as the spatial extent of a resource affects the costs of defining reasonable limits and therefore of monitoring them over time.

Given the activity development and short-term practice it cannot be stated that such a scenario would lead *lagartos* population to its extermination or if, otherwise, the hunters would develop defense mechanisms and control over time. Some access and lax regulation rules like “don’t destroy caves” or “don’t hunt small animals” were reported as defined by hunters and markets, respectively.

Resource depletion occurs when the demand exceeds the resource capacity for self-sustaining and technologies exist to exploit resources at high levels [53]. As evidence suggests high levels of *lagarto* exploitation in the region lack the technology to exploit the resource (e.g. motor boats), and environment restrictions and the vastness of the wetland may particularly have functioned as obstacles to a potential over-exploitation. Hunters stated that not all sites could be exploited because of the difficulty in accessing them, which in turn indirectly leads to the creation of intangible zones that could serve as breeding areas or “sources” for the already exploited areas each year [56].

On the other hand when resources are relatively abundant, there is little reason for users to invest time and effort in organizing the activity [47]. Although *lagartos* were abundant, in these contexts Mayan hunters still developed a system of socio-spatial management. But why do they do it? Above all, we argue that this system of cooperation promoted the distribution of benefits among groups of hunters. This is in agreement with that reported by Berkes [54]. He found that where areas to be defended are large, some system of cooperation and reciprocal use rights may develop with adjacent territory-holders, as it happened with hunting territories in the James Bay area [54]. However, differently from that reported by the latter for the territories in his studies, the *rumbos* and *trabajaderos* defined and used by the Mayan, represented areas of use not socially validated as the “ownership” of hunters groups. Instead, this arrangement ensured more or less successful harvests.

## Conclusions

The analysis of *lagartos* hunt practiced in the past by the Mayan of *Xhazil*, allowed the identification of factors and mechanisms involved in the emergence of a new activity. In this way we can better understand the various ways in which human groups face change and uncertainty.

As we have been discussing, we validate our initial hypothesis about the development and accomplishment of a new activity by the Mayan of *Xhazil*. On the one hand, they used their traditional social organization structure as well as their culturally inherited knowledge. On the other hand, they acquired new ecological knowledge of the species in a learning-by-doing process, together with the use of other sources of external information.

We noted that although the activity was developed on open tenure lands, we identified some of the guiding principles of a management system such as social and spatial organization, and traces of certain norms and rules of use. The system described is consistent with the “ways of doing” of these Mayans but is shaped by the resource characteristics and the constraints imposed by the *savanna*.

Finally we consider results of this research contribute to the discussion of important issues such as continuity of traditional knowledge, resource management and conservation of land and resources that sustain Mayan life in the Yucatan Peninsula of México. In turn, this study highlights the importance of considering social and cultural structures in the development of management plans and new production activities in local areas.

## Abbreviations

UNC: Universidad Nacional de Córdoba, Argentina; ECOSUR: El Colegio de la Frontera Sur Chiapas Mexico; IBS: Instituto de Biología Subtropical, Universidad Nacional de Misiones, Argentina; UIA: Universidad Iberoamericana (UIA).

## Competing interests

The authors declare that they have no competing interests.

## Authors’ contributions

FZ designed and coordinated the study, performed the field survey, carried out the analyses and prepared and drafted the manuscript. EBB and EEL made substantial contributions to theoretical background, conception and design of the study, field work, data analysis and interpretation of results. All authors read and approved the final manuscript.

## Authors’ information

FZ. Biologist at the University of Cordoba (UNC), Argentina, MSc in Natural Resources and Rural Development at El Colegio de la Frontera Sur (ECOSUR) Mexico and PhD at UNC, Argentina. Currently independent researcher at the Institute of Subtropical Biology (IBS) in Puerto Iguazu, Argentina. EBB. Agricultural and PHD in Social Anthropology at the Universidad Iberoamericana (UIA). Researcher in the Department of Agriculture, Society and Environment of El Colegio de la Frontera Sur, San Cristobal de las Casas (Chiapas) México. EIJEL. Biologist and PhD in Social Anthropology at the Universidad Iberoamericana (UIA). Researcher in the Department of Agriculture, Society and Environment of El Colegio de la Frontera Sur, San Cristobal de las Casas (Chiapas) México.
